# Supplementary material for: Content-rich biological network constructed by mining PubMed abstracts
Source: BMC Bioinformatics. 2004 Oct 8;5:147. doi: 10.1186/1471-2105-5-147 (PMC528731; doi:10.1186/1471-2105-5-147)
Supplement: Additional File 2 — The original results of the above study (non-essential files are deleted to keep the file size under the limit set by BMC bioinformatics). [file 1471-2105-5-147-S2.bz2 › chilibotAdditionalFile2/dip05/164ID8702683E570/html/GHR_STAT5A.html]

 


 **GHR** and **STAT5A** 
  
Found 75 abstracts in PubMed, retrieved 05.  
 

 What does Google say? 
 PDF only 
| .edu only 

---

**Interactive relationship** (e.g. stimulation, inhibition, etc)

**Non-interactive relationship** (e.g. studied together, co-existance, homology, etc.)

- The effect of heterodimerization was studied by comparing activation of Janus kinase 2, signal transducer and activator of transcription STAT 1, STAT3, STAT5  [ **STAT5A** ] , and MAPK in Chinese hamster ovary cells stably transfected with chimeric genes encoding receptors consisting of cytosolic and transmembrane parts of oGHR and oPRLR, extracellular domains of human granulocyte and macrophage colony stimulating factor hGM CSF receptor alpha or beta, and cells transfected with the two forms alpha or beta of PRLR and  **GHR** .  Ref: 12865335 Endocrinology, 2003
- In C2C12  **GHR**  cells, we observed an increased activation of the Janus kinase 2 Jak2, signal transducers and activator of transcription 5 Stat5  [ **STAT5A** ] , mitogen activated protein kinase MAPK and protein kinase B Akt upon acute GH stimulation.  Ref: 12681449 Cell Signal, 2003
- STAT5  [ **STAT5A** ] , STAT3,  **GHR** , Socs 1 3, and Cis phosphorylation and abundance were assessed by using immunoblots, EMSA, and or real time RT PCR.  Ref: 12519742 Am J Physiol Gastrointest Liver Physiol, 2003
